# Intracranial hemorrhage in a patient with depressive anxiety disorder about a case

**DOI:** 10.1192/j.eurpsy.2023.1952

**Published:** 2023-07-19

**Authors:** M. Palomo Monge, M. V. Lopez Rodrigo, C. Garcia Montero, A. Osca Oliver, V. R. Fons, A. Duque Dominguez

**Affiliations:** 1 Psychiatry, hospital nuestra señora del prado, Talavera de la Reina; 2 Psychiatry, hospital provincial de Ávila, Ávila, Spain

## Abstract

**Introduction:**

We present the case of a 69-year-old patient who went to the emergency department due to an episode of aggressiveness and behavioral alteration, presenting irritability and nervousness, of about 2 days of evolution according to her family member. Given that the patient had previously presented chronic behavioral disorders and had previously been followed up in psychiatric consultations, psychiatry was notified after an initial evaluation by the emergency physician.

**Objectives:**

Somatic personal history: NAMC. HTA. Not DM, not DL. Former smoker of 20 cigarettes/day. Recurrent intracranial hemorrhage secondary to amyloid angiopathy and suspected amyloid vasculitis. Last admission to the neurology service in June 2022, also presenting symptomatic epileptic seizures and secondary behavioral alterations. Mastocytosis. Post-traumatic vertebral fracture. Non-anticoagulated paroxysmal atrial fibrillation. Surgical: Left ear surgery. appendectomy. Hysterectomy + oophorectomy.

Personal psychiatric history: In follow-up since May 2021 referred from neurology for emotional lability, episodes of anger and fear. Diagnosed with anxiety-depressive disorder secondary to a medical illness.

Current psychiatric treatment: Oxcarbazepine 800mg 0-0-1, trazodone 100mg 0-0-1, aripiprazole 10mg 1-0-0.

**Methods:**

Current illness: The patient goes to the emergency room brought by her husband. During the interview she minimizes her aggressive behaviors or even does not remember them. She is disoriented in time, with very striking memory failures. Her husband comments verbal aggressiveness if he contradicts her in something, sometimes even presenting physical aggressiveness with her relatives. They report that in the last psychiatric consultation a little over 1 month ago, aripiprazole was withdrawn due to an increased risk of cardiovascular events.

After the examination of the patient, she was referred back to the emergency department for a new assessment and to rule out the organicity of the current condition, given that the patient had cardiovascular risk factors, due to the suspicion of a new episode of intracranial hemorrhage.

**Results:**

sychopathological examination: Vigil, conscious, disoriented in time, partially in space. Collaborative, calm during the interview. Coherent, structured speech, with obvious memory failures. Labile, irritable mood. Verbal and physical heteroaggressiveness at home, not during the interview. No structured or planned autolytic ideation at this time. Appetite and sleep preserved.

**Conclusions:**

An urgent head CT was requested, with the result of a small intraparenchymal bleeding in the left frontal location, and she was admitted to the neurology department, with a diagnosis at discharge of: small left frontal haematoma, suspected amyloid vasculitis, and secondary behavioral alteration (vascular dementia).

**Disclosure of Interest:**

None Declared

